# Detection of *ERBB2* (HER2) Gene Amplification Events in Cell-Free DNA and Response to Anti-HER2 Agents in a Large Asian Cancer Patient Cohort

**DOI:** 10.3389/fonc.2019.00212

**Published:** 2019-04-04

**Authors:** Jeeyun Lee, Aleksandra Franovic, Yukimasa Shiotsu, Seung Tae Kim, Kyoung-Mee Kim, Kimberly C. Banks, Victoria M. Raymond, Richard B. Lanman

**Affiliations:** ^1^Division of Hematology-Oncology, Department of Medicine, Samsung Medical Center, Sungkyunkwan University of Medicine, Seoul, South Korea; ^2^Department of Medical Affairs, Guardant Health Inc., Redwood City, CA, United States; ^3^Department of Pathology and Translational Genomics, Samsung Medical Center, Sungkyunkwan University School of Medicine, Seoul, South Korea

**Keywords:** cfDNA, ERBB2, HER2, liquid biopsy, NGS

## Abstract

**Background:** HER2 antagonists have marked activity and are approved for the treatment of HER2 overexpressing breast and gastric cancers. Recent studies have shown that *ERBB2* (HER2) gene amplification and overexpression may also be actionable in other tumor types. Inter- and intratumoral heterogeneity in HER2 status, however, poses a significant challenge in identifying patients that may benefit from HER2-targeted therapies. *ERBB2* amplification as identified by circulating cell-free DNA (cfDNA), which circumvents tissue heterogeneity issues, is emerging as a robust biomarker predictive of response to anti-HER2 agents. Here, the prevalence and genomic landscape of *ERBB2* alterations detectable by next-generation sequencing (NGS) of cfDNA was evaluated in a large cohort of Asian patients with advanced solid tumors.

**Methods:** Results were queried for consecutive patients (*n* = 469) tested by a comprehensive 70/73-gene cfDNA NGS assay (Guardant360®) between November 2015 and June 2018. Patients with *ERBB2* gene alterations including copy number amplifications (CNAs), single nucleotide variants (SNVs), and insertion-deletions (indels) were identified.

**Results:** *ERBB2* alterations were detected in 52 patients (11.1%); *ERBB2* SNVs, CNAs, and indels were found in 27 (5.8%), 27 (5.8%), and 10 (2.1%) patients, respectively. *ERBB2* amplification was most frequently identified in gastric (21.4%; 6/28), colorectal (11.1%; 5/45), lung (3.9%; 9/231), and breast (3.2%; 1/31) cancer patients. *ERBB2* amplification was often mutually exclusive with other oncogenic alterations in gastric (83.3%; 5/6) and colorectal (60%; 3/5) cancer patients. *ERBB2* copy number gains were also highest in gastric and colorectal cancers (median 4.8 and 6.6, respectively). We further report two cases of advanced gastric cancer patients, one treatment naïve, and the other having failed four lines of therapy, whose *ERBB2* CNAs were identified by cfDNA and derived clinical benefit from HER2-based therapies.

**Conclusion:** Our data indicate that *ERBB2* amplification is a common event in solid tumors among Asian cancer patients. High *ERBB2* incidence and copy number gains were observed in gastric and colorectal cancer patients, often in the absence of other oncogenic mutations, underscoring its likely role as the driver alteration in those settings. Finally, we show the potential of comprehensive cfDNA testing in identifying patients who are most likely to benefit from HER2-targeted therapies.

## Introduction

The human epidermal growth factor receptor-2 (HER2) is a receptor tyrosine kinase, belonging to the ErbB family (EGFR/HER1, HER2, HER3, and HER4), involved in signal transduction pathways that mediate key cellular processes including cell proliferation, differentiation, and survival (1). HER2 deregulation via gene mutation, amplification, and post-transcriptional upregulation has been observed in a wide array of human cancers. Importantly, it has been well-established that its aberrant expression and activation is sufficient to drive cell transformation and oncogenesis in preclinical models; an observation consistent with clinical and etiological findings in certain cancer indications (2, 3).

Among the clinical evidence supporting its oncogenic driver role, HER2 antagonists have had demonstrable clinical activity in patients with HER2 overexpressing breast and gastric cancers. Clinical trials evaluating trastuzumab, a monoclonal antibody targeting HER2, when combined with chemotherapy in patients with HER2-positive breast and gastric cancers yielded objective response rates (ORR) of ~50% and median overall survivals (mOS) surpassing 1–2 years (4, 5). More recently it has been shown that addition of pertuzumab, an antibody targeting a different HER2 epitope, to trastuzumab/chemotherapy increased ORR to 80% and mOS to 56.5 months in patients with metastatic breast cancer (6, 7). Based on these encouraging results, HER2 therapies have been approved in the first-line setting for the treatment of metastatic breast and gastric cancer patients.

Similarly, impressive response rates and clinical benefit have been reported in clinical trials evaluating anti-HER2 agents in patients with heavily pretreated HER2-positive colorectal cancer (CRC). Among 33 patients enrolled in the phase II HERACLES trial testing trastuzumab and lapatinib, a small molecule HER2/EGFR tyrosine kinase inhibitor, in patients with *ERBB2* (HER2) amplified/*KRAS* wild-type CRC, the ORR was 30% with disease stabilization achieved in 70% of patients (8). Importantly, the observed responses were durable; half of the patients remained on therapy for more than 6 months and one patient for over 3 years (9). Notably, patients with higher tissue *ERBB2* copy numbers (CN; ≥10 copies) derived the most clinical benefit and had a significantly longer time to progression compared to those with lower copy number gains (53.1 vs. 34 weeks, respectively). These findings compare favorably to the response rates observed with immunotherapy (ORR 35%) in patients with treatment-refractory CRC and underscore *ERBB2* gene amplification and overexpression as a targetable oncogenic driver in advanced CRC (10, 11).

Historically, *ERBB2* gene amplification and protein overexpression in tumor biopsy material, as measured by *in situ* hybridization (ISH) or immunohistochemistry (IHC), has been used to select patients most likely to benefit from HER2-based therapeutic strategies. A major challenge with this approach is the described inter- and intratumoral heterogeneity in HER2 status, particularly in cancers of gastrointestinal (GI) origin and breast cancers, due to tissue sampling bias. *ERBB2* gene amplification identified by next-generation sequencing (NGS) of tissue DNA or cell-free DNA (cfDNA) in the blood is emerging as a robust biomarker predictive of response to HER2 targeted agents and offers an alternative to IHC/ISH (12). We recently reported that among a cohort of HER2-positive gastric cancer (GC) patients treated with a combination of lapatinib and capecitabine/oxaliplatin, the response rate for those with detectable *ERBB2* copy number amplification in their blood was 100% (12, 13). Moreover, it has been shown that cfDNA captures tumor heterogeneity in GC patients, allowing for the identification of the 10–20% HER2-negative primary tumors with synchronous HER2-positive metastatic lesions (12, 14–17).

Here, we examined the prevalence and genomic landscape of potentially actionable *ERBB2* gene amplification-positive advanced solid tumors as detected by NGS of cfDNA in a large cohort of patients from Asia. We further present case reports of patients with metastatic and treatment-refractory GC that had meaningful clinical responses to HER2-based therapies initiated upon detection of *ERBB2* gene amplification by cfDNA testing.

## Methods

### cfDNA Sequencing Platform

The Guardant360® panel is a CLIA-certified, College of American Pathologists (CAP)-accredited, New York State Department of Health (NYSDOH)-approved test that detects single nucleotide variants (SNVs) (70 or 73 genes), copy number amplifications (CNAs) (18 genes), insertion-deletion alterations (indels) (23 genes), and fusions (6 genes). Briefly, cfDNA is barcoded for digital sequencing library preparation. This library is amplified and enriched for the target genes using biotinylated custom baits. Each of the cancer-related genes is paired-end sequenced on an Illumina NextSeq 500 and/or HiSeq 2,500 at a 15,000x average coverage depth per base pair. After sequencing, algorithmic reconstruction of the digitized sequencing signals is used to reconstruct the cfDNA fragments.

The absolute number of unique DNA fragments at a given nucleotide position is quantified, enabling measurement of circulating tumor DNA (ctDNA) as a percentage of the total cfDNA. The mutant allele frequency (MAF) for a given somatic alteration is calculated as the fraction of cfDNA molecules harboring the variant of interest divided by the total number of unique cfDNA molecules mapping to the variant position. The reportable range for SNVs, indels, fusions, and CNAs in cfDNA by the Guardant360 assay is ≥0.04%, ≥0.02%, ≥0.04%, and ≥2.12 copies, respectively. Plasma copy number is reported by centiles with 2+ being between the 50th to 90th-centile in the Guardant Health database and 3+ being greater than the 90th-centile. Focal gene amplifications are determined bioinformatically as those with statistically higher copy numbers relative to other genes across the same chromosome arm.

Analytic and clinical validation of the assay has been previously reported (18, 19).

### cfDNA *ERBB2* Landscape

The Guardant Health clinical results database was queried for consecutive ctDNA-positive patient (*n* = 469) samples originating from 15 Asian centers tested between November 2015 and June 2018 (70- or 73-gene panel versions). Patients with non-synonymous *ERBB2* gene alterations (CNA, SNV, and indel) were identified. The prevalence of *ERBB2* alterations among patients with GI (*n* = 129) and non-GI (*n* = 340) cancers were compared. The cfDNA genomic landscape of patients exhibiting *ERBB2* amplification was further assessed. Variants of unknown significance were excluded from the analysis of co-occurring alterations. In addition, the prevalence of *ERBB2* amplification and copy number gains by cancer type (gastric, breast, colorectal, pancreatic, and lung cancer) was compared between Asian and non-Asian patient cohorts. The latter cohort included ctDNA-positive patient samples received from non-Asian centers and tested during the same time period (*n* = 31,412 patients) This research was conducted as per a protocol approved by the Quorum Institutional Review Board (IRB) for the generation of de-identified data sets for research purposes.

### Statistical Analyses

A two-tailed Student *t*-test was applied to determine the difference between the means of two groups. Population proportions were evaluated using a z-score test to determine the associated *P*-values.

## Results

### cfDNA and *ERBB2* Gene Alteration Detection in Asian Patient Cohort

**Figure 1:**
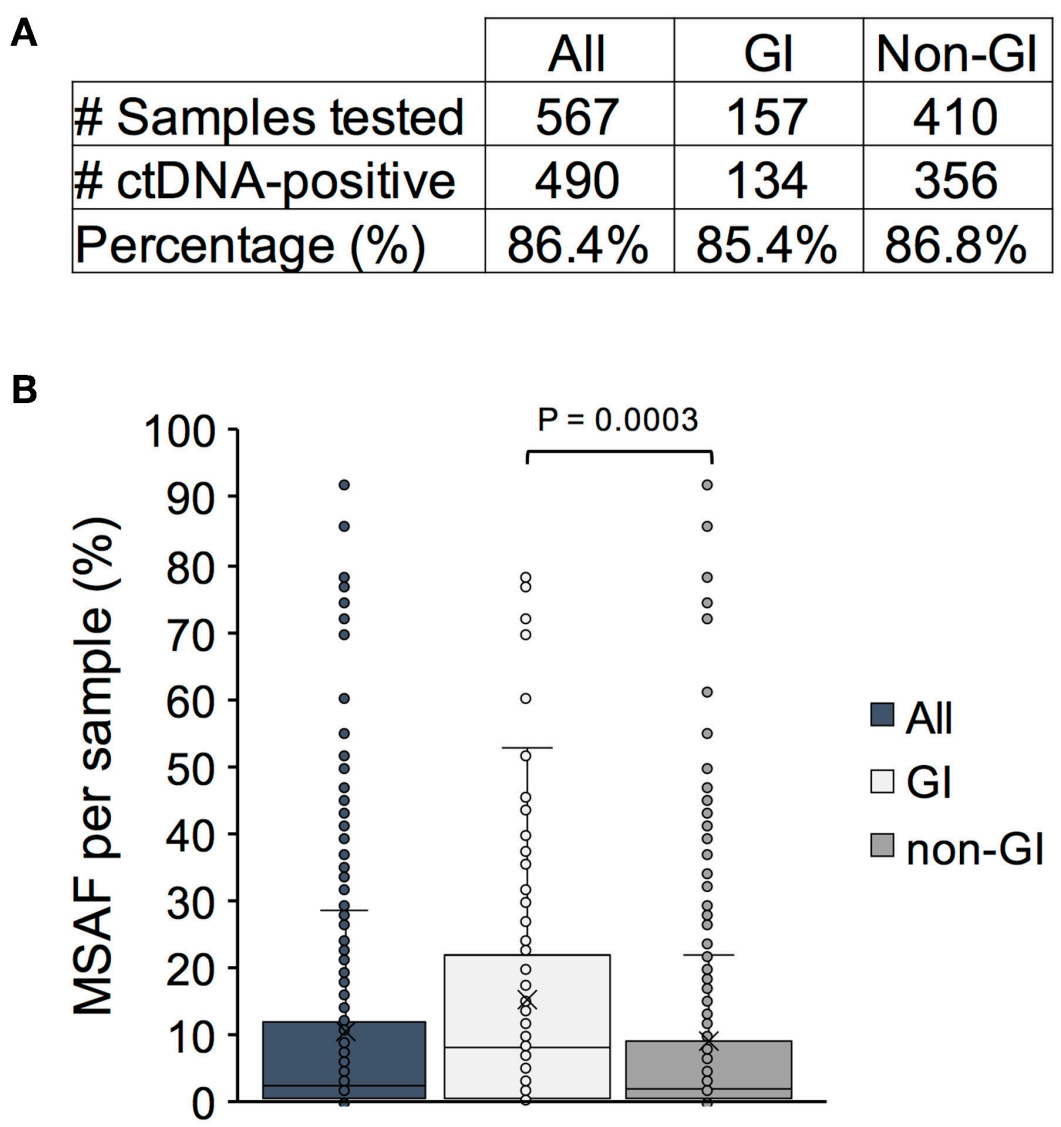
Detection of circulating tumor DNA (ctDNA) and degree of DNA shedding as measured by maximum somatic mutant allele fraction (MSAF) in an Asian patient cohort. **(A)** The number and percentage of ctDNA-positive cancer patient samples in the entire cohort vs. a subset of patients with gastrointestinal (GI) and non-GI tumors. **(B)** The MSAF per patient sample plotted for the entire cohort and by indication. The mean and median values are denoted by an x and a bar, respectively.

The Guardant Health clinical database was queried for Asian patients that had received Guardant360 cfDNA testing between November 2015 and June 2018 (*n* = 567 samples; *n* = 539 unique patients). Patient samples were received from Hong Kong, Singapore, Japan, Thailand, South Korea, and Taiwan (Supplementary Figure 1). The average age in this patient cohort was 60 (range 26–116) with equivalent gender representation (53% female/47% male; Supplementary Table 1). Of the samples tested, ctDNA was detected in 86.4% (490/567; Figure 1A). Similar rates of ctDNA detection were observed among patients with GI (listed in Supplementary Table 1) and non-GI cancers (85.4 vs. 86.8%; *P* = 0.664).

We next examined the maximum somatic mutant allele frequency (MSAF) identified per sample which can serve as a proxy for the degree of tumor DNA shedding into circulation. The median MSAF across all SNV, indel, and fusion variants detected in patient samples was 2.7% (range 0.03–92%; Figure 1B). A wide range of values has been previously reported and can be attributed to various biological factors affecting ctDNA shedding including, but not limited to, the degree of metastatic disease burden and proximity to the vasculature (19, 20). A significantly higher median MSAF was observed in patients with GI cancers (7.9%; range 0.1–78%) compared to those with non-GI cancers (1.8%; range 0.03–92) (*P* = 0.0003). This finding is consistent with prior reports indicating that GI tumors are high shedders and thus optimal candidates for cfDNA testing (21).

### *ERBB2* Gene Amplification Prevalence in Asian GI Patients

**Figure 2:**
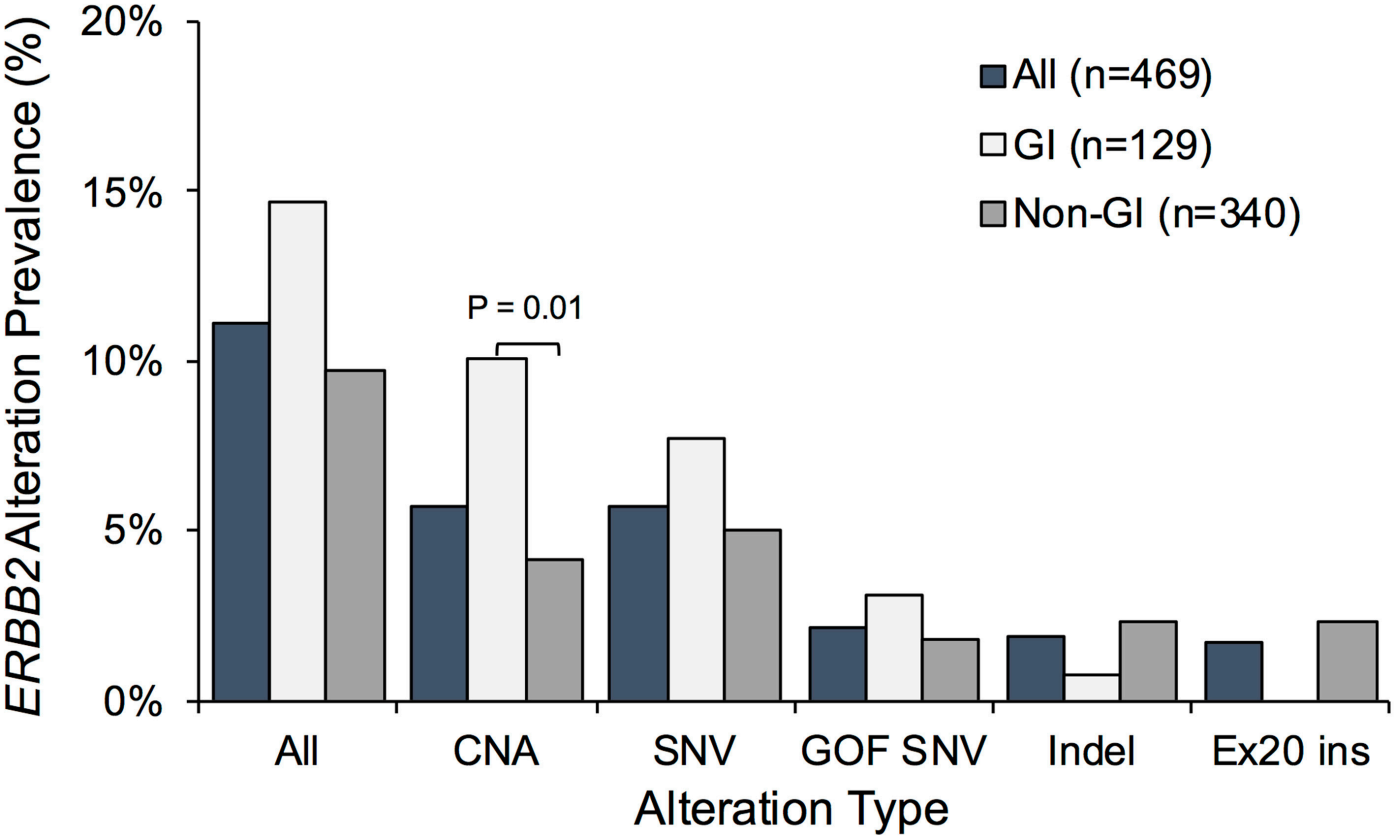
Prevalence of non-synonymous *ERBB2* gene alterations identified in cell-free DNA in the full Asian patient cohort vs. patients with gastrointestinal (GI) or non-GI cancers. Percentage of patients exhibiting *ERBB2* copy number amplification (CNA), single nucleotide variants (SNV), and/or insertion deletion variants (indels) was plotted. Patients with gain-of-function (GOF) SNVs and activating exon 20 insertions (Ex20 ins) represent subsets of those with SNVs and indels, respectively.

Non-synonymous *ERBB2* alterations were detected in 52 of the 469 ctDNA-positive patients (11.1%) (Figure 2); *ERBB2* CNA, SNV, and indel mutations were found in 27 (5.8%), 27 (5.8%), and 9 (1.9%) patients, respectively. Activating *ERBB2* gene mutations including gain-of-function SNVs and exon 20 insertions were observed in 2.1% (10/469) and 1.7% (8/469) of patients. While the prevalence of *ERBB2* amplification was higher in patients with GI cancers (*P* = 0.013), no exon 20 insertion mutations were observed in that subset of patients but rather were exclusively identified in patients with lung cancer (Figure 2).

**Figure 3:**
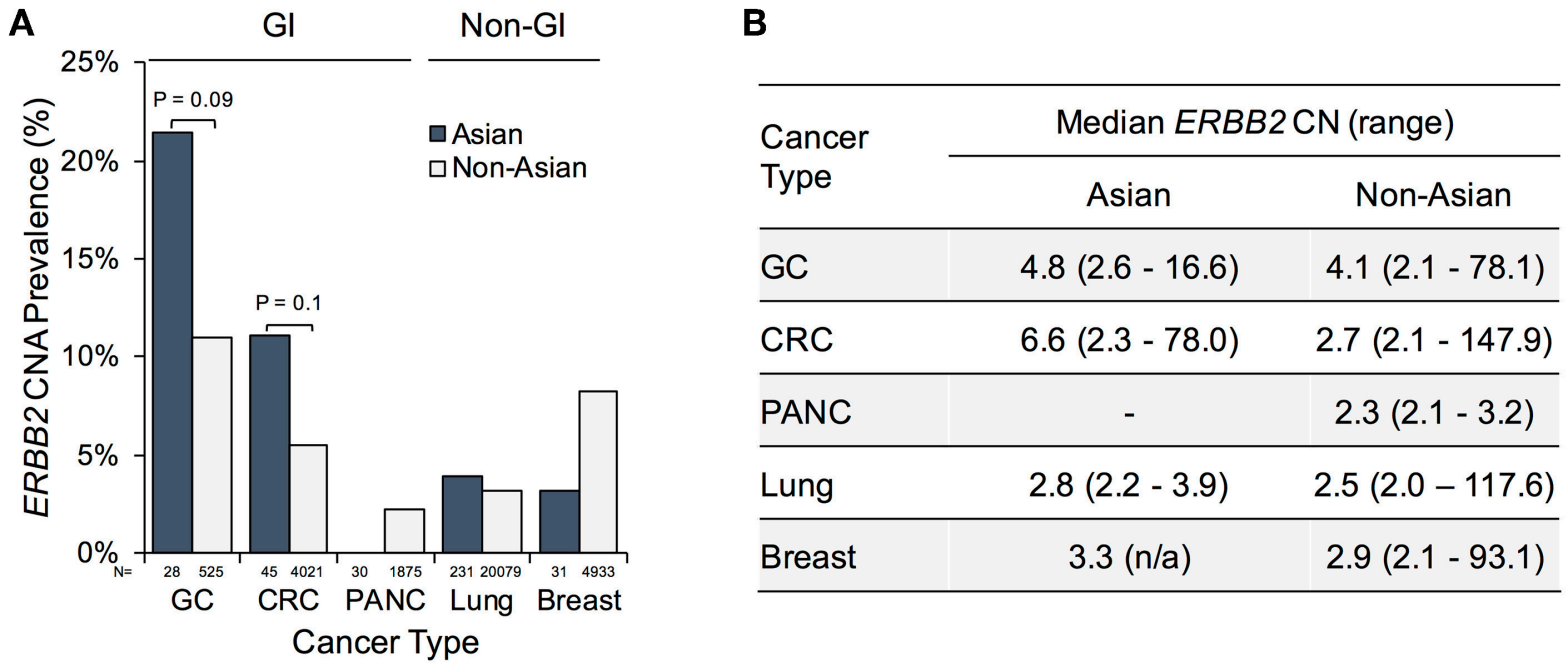
*ERBB2* gene amplification prevalence and copy number gains in Asian vs. non-Asian patients by cancer type. **(A)** Percentage of patients exhibiting *ERBB2* gene amplification as identified in cell-free DNA. Total patient numbers tested per indication listed below bars. **(B)** Median blood *ERBB2* copy number and range among patients with *ERBB2* gene amplification. GI, gastrointestinal; GC, gastric cancer; CRC, colorectal cancer; PANC, pancreatic cancer; CN, copy number.

*ERBB2* gene amplification was most frequently identified in patients with gastric (21.4%; 6/28), colorectal (11.1%; 5/45), lung (3.9%; 9/231), and breast (3.2%; 1/31) cancers (Figure 3A, Supplementary Figure 2). The *ERBB2* gene amplification prevalence observed in Asian patients with GC and CRC exceeded that observed in non-Asian patients (11% [58/525; *P* = 0.093] and 5.5% [223/4021; *P* = 0.103], respectively). In contrast, the frequency of *ERBB2* gene amplification in pancreatic, lung, and breast cancers was similar or lower compared to non-Asian patients. The absolute *ERBB2* copy number in plasma was equivalent among Asian and non-Asian gastric (median CN = 4.8 vs. 4.1), lung (median CN 2.8 vs. 2.5), and breast (median CN 3.3 vs. 2.9) cancer patients but higher in Asian CRC patients (median CN 6.6 vs. 2.7; *P* = 0.146; Figure 3B). Notably, the observed plasma *ERBB2* CNs were higher in GI compared to non-GI cancers, indicating that they may be more biologically relevant in these disease settings.

### Detection of Focal vs. Aneuploidy-Related *ERBB2* Copy Number Gains

**Figure 4:**
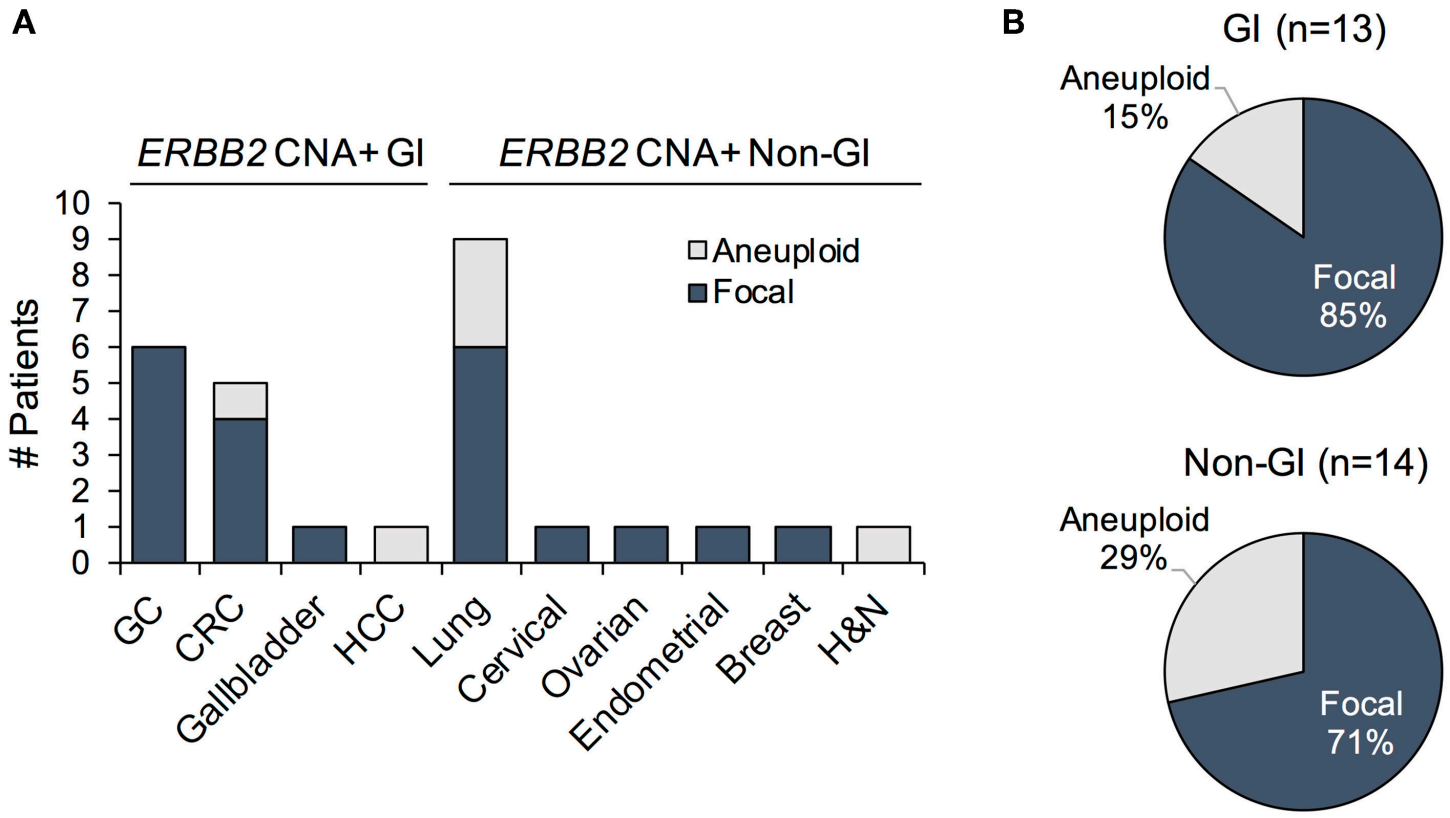
**(A)** Prevalence of patients exhibiting *ERBB2* gene amplification with focal vs. aneuploidy-related copy number gains plotted by cancer type. **(B)** Proportion of focal vs. chromosome/arm level *ERBB2* amplification events among *ERBB2*-amplified gastrointestinal (GI) and non-GI cancer patients. GC, gastric cancer; CRC, colorectal cancer; HCC, hepatocellular cancer; H&N, head and neck cancer.

*ERBB2* gene amplifications can be focal or may be due to chromosome/arm level copy number gains. While the clinical implications of these different mechanisms have not been thoroughly probed, studies indicate that focal amplifications typically affect candidate oncogenic drivers (22). Thus, patients harboring focal *ERBB2* CNAs may be more likely to respond to its therapeutic targeting. Unlike IHC/ISH, NGS testing is capable of distinguishing focal gene amplification events from aneuploidy-associated copy number gains. We therefore reanalyzed the samples to establish the incidence of focal *ERBB2* gene amplifications, as identified in cfDNA, in this patient cohort. All of the GC (6/6) and 80% (4/5) of CRC *ERBB2* CNAs were focal (Figure 4A). Most of the non-focal calls were observed in patients with lung cancer, which exhibited more simultaneous gene CNAs on average compared to other cancer types (median 4.5 vs. 3, respectively) indicative of aneuploidy (Figure 4A, Supplementary Table 2). The majority of *ERBB2* copy number gains across cancer types, however, were in fact focal; accounting for 85 and 71% of calls in GI and non-GI cancers, respectively (Figure 4B). cfDNA genomic landscape in *ERBB2*-amplification positive GI patient samples

**Figure 5:**
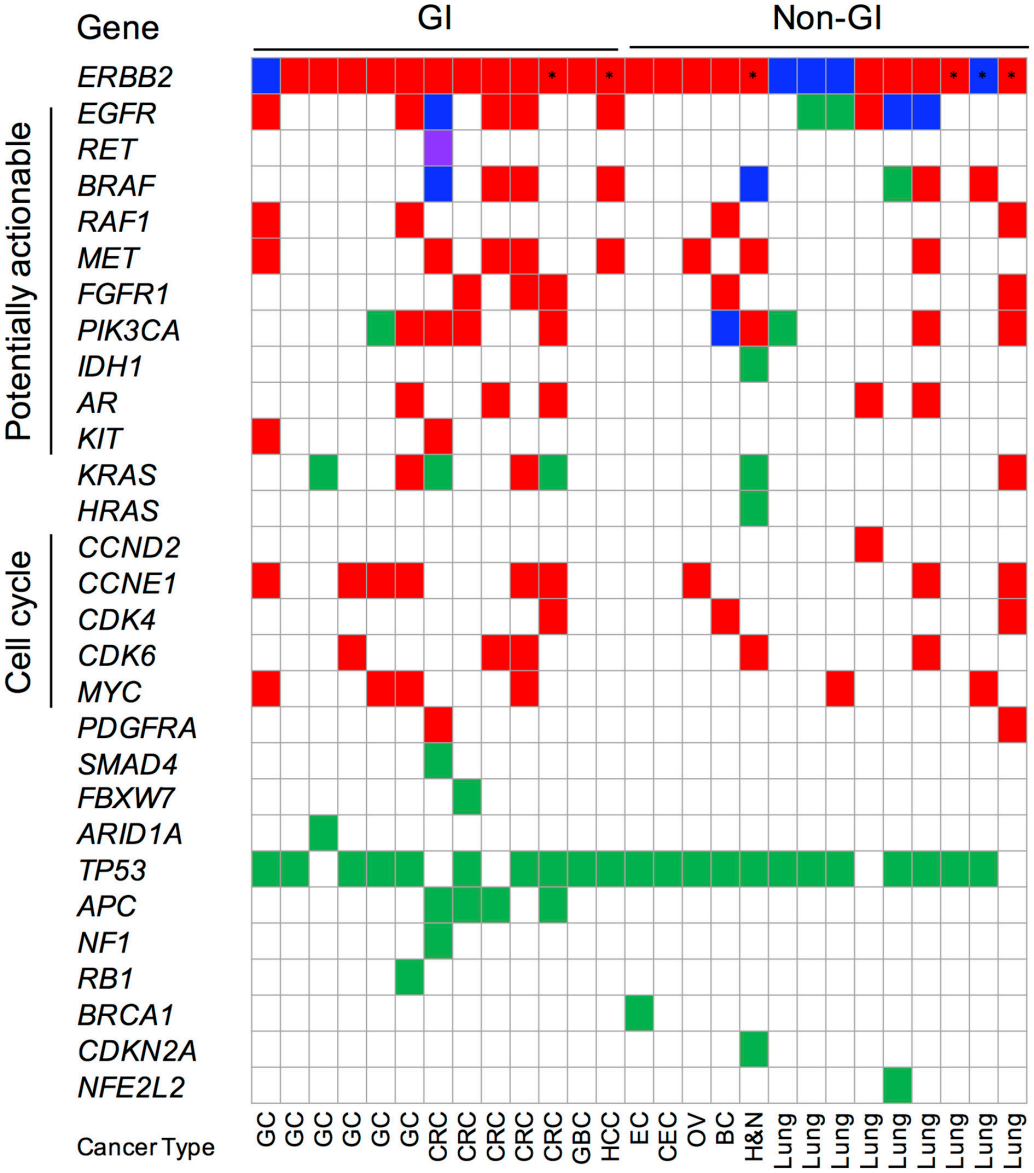
Oncoprint of cfDNA genomic landscape of *ERBB2*-amplified gastrointestinal (GI) and non-GI Asian cancer patients. Red fill denotes copy number amplification (CNA); green fill denotes single nucleotide (SNV) or insertion-deletion variants; blue fill denotes concurrent CNA and SNV; purple fill denotes gene fusions; and asterisk denotes patients with aneuploidy-related *ERBB2* CNA events. Synonymous alterations and variants of unknown significance excluded from analysis. GC, gastric cancer; CRC, colorectal cancer; GBC, gallbladder cancer; EC, endometrial cancer; CEC, cervical cancer; OV, ovarian cancer; BC, breast cancer; H&N, head and neck cancer.

In addition to focality, the mutual exclusivity with other driver mutations is indicative of a more significant role for an amplified gene in cancer development and progression. *ERBB2* amplification was often mutually exclusive with canonical oncogenic alterations in GC (83.3%; 5/6) and CRC (60%; 3/5) cancer patients, reinforcing its likely importance in these cancer types (Figure 5, Supplementary Table 2). In contrast, co-occurring oncogenic mutations, including *BRAF* V600E, *EGFR* L858R/exon 19 deletion, multiple *K/HRAS* G12/13 variants, and *PIK3CA* E542K/E545K, were detected in 50% (7/14) of *ERBB2* amplified non-GI cancers (Figure 5, Supplementary Table 2). Consistent with the reported prevalence in Asian patients, 44.4% (4/9) of the lung cancer patients exhibited activating *EGFR* mutations.

Copy number gains in multiple genes accounted for the majority of co-occurring somatic variants identified in both *ERBB2*-amplified GC (66.6%; 4/6) and CRC (100%; 5/5) patient samples (Figure 5). Co-occurring *CCNE1* gene amplification was observed in 66.6% (4/6) of GC while potentially actionable receptor tyrosine kinase gene (*EGFR, MET*, and *FGFR1*) copy number gains were observed in 100% (5/5) CRC patient samples. The latter finding suggests that combination therapies geared at targeted both EGFR and HER2 (i.e., trastuzumab + cetuximab or lapatinib) may be beneficial in CRC that display amplification of both genes.

### Case Reports

To assess the reliability of cfDNA testing in correctly identifying actionable *ERBB2* gene amplifications, in the absence of co-occurring mutations that may impact therapeutic response, the following case studies were considered.

**Figure 6:**
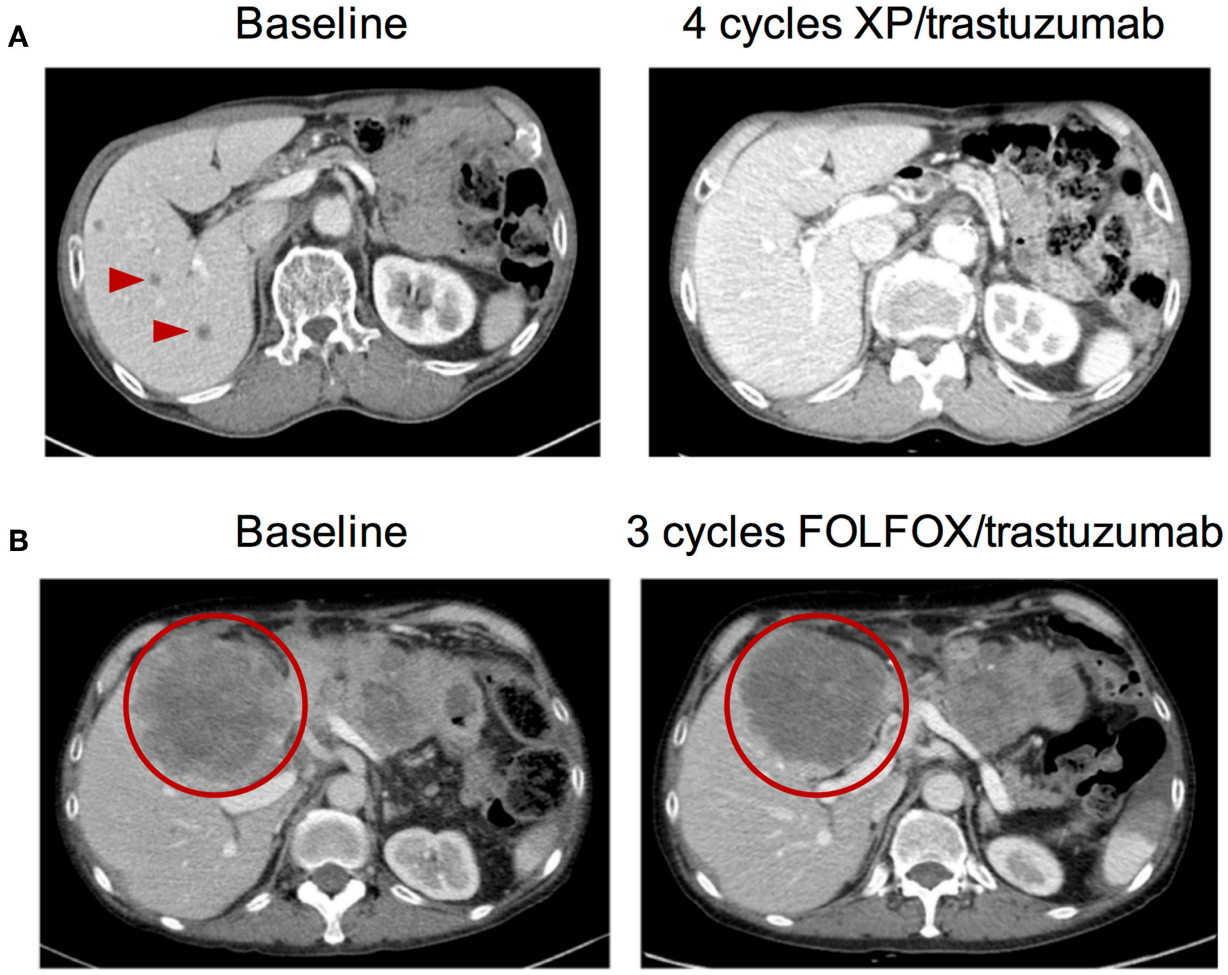
Pre- and post-anti-HER2 therapy abdominal CT images from two *ERBB2*-amplified gastric cancer patients identified by cell-free DNA. **(A)** Patient #1 at baseline and after four cycles of capecitabine/cisplatin (XP) combined with trastuzumab. **(B)** Patient #2 at baseline and after three cycles of FOLFOX combined with trastuzumab. Arrowheads and circles denote malignant lesions.

Patient #1—A 70-year old male was diagnosed with advanced gastric cancer with multiple liver metastases. The pathologic examination revealed that the tumor was a tubular adenocarcinoma with moderate differentiation in grade. The HER2 IHC of the primary tumor showed HER2 overexpression (3+) in all tumor areas examined. Blood-based cfDNA genomic profiling was simultaneously performed using the Guardant360 73-gene panel. Consistent with the IHC result, the Guardant360 test demonstrated a focal *ERBB2* gene amplification of 15.47 copies in plasma (3+) in the absence of any other oncogenic variants. As a result, the patient was subsequently treated with capecitabine/cisplatin (XP) in addition to trastuzumab. After two treatment cycles, the liver metastases, and gastric cancer decreased in size by >80% from baseline per RECIST criteria which was maintained for eight cycles of XP/trastuzumab. Multiple liver metastases had nearly disappeared within four cycles of XP/trastuzumab (Figure 6A).

Patient #2—A 56-year old male initially diagnosed with gastric cancer was treated and experienced disease progression after 12 cycles of FOLFIRI, four cycles of docetaxel, four cycles of nivolumab, and four cycles of taxol/ramucirumab. There was no evidence of *ERBB2* amplification or HER2 overexpression in the primary tumor specimen taken at the time of diagnosis; tissue genotyping demonstrated only *CCNE1* gene amplification when DNA was extracted from baseline tumor biopsy. During treatment with taxol/ramucirumab, the patient received Guardant360 cfDNA profiling which revealed a high level focal *ERBB2* gene amplification (4.75 copies in the blood; 3+). Consistent with the primary tissue biopsy result, a *CCNE1* gene amplification was also detected by cfDNA analysis. By the time the *ERBB2* amplification was identified, the patient had disseminated metastases to the liver, peritoneum, lung, and brain. The patient received five cycles of FOLFOX/trastuzumab which stabilized the disease for 3 months with significantly reduced cancer pain which was indicated by reduced opioid medication following chemotherapy (Figure 6B). Unfortunately, the patient died of aspiration pneumonia.

## Discussion

While it is well-appreciated that *ERBB2* (HER2) gene amplification and protein expression is a bona fide therapeutic target in the treatment of patients with breast and gastric cancers, more recent trials have drawn attention to its actionability in other cancer types (23, 24). We report here that focal *ERBB2* gene amplification, as identified by cfDNA analysis, is common in various solid tumor types in a large cohort of patients from Asia. Strikingly, the prevalence of *ERBB2* gene amplification in Asian GC (21.4 vs. 11%) and CRC (11.1 vs. 5.5%) patients is higher than in non-Asian patient samples (Figure 3A). The former observation is consistent with a large study that identified HER2 overexpression, by IHC/ISH, in 9.7% of all Asian GC patients (*n* = 5,301) and 18.1% when China was excluded from the analysis, suggesting regional differences in prevalence (25). In the case of primary CRC, however, another study reported an *ERBB2* amplification/overexpression frequency of only 3.3% in an Asian patient cohort (*n* = 4913). A potential explanation for the divergent findings is that the patient population reported here is biased toward heavily pre-treated patients such that they may have acquired *ERBB2* gene amplification during the course of disease progression or as a mechanism of resistance to prior therapies (26). Interestingly, the absolute *ERBB2* gene copy number gains as measured in plasma are highest in GI cancers, in particular in Asian CRC patients (median CN 6.6 vs. 2.7 in non-Asian patients; Figure 3B). While additional studies in larger Asian patient cohorts would be required to corroborate these findings and achieve statistical significance, they clearly demonstrate that a substantial portion of GC and CRC cancer patients present with or acquire high-level focal *ERBB2* amplification.

Further supporting a pathogenic role for HER2 in patients with GC and CRC is the finding that *ERBB2* gene amplification is rarely detected in conjunction with a canonical oncogenic mutation (Figure 5). Only 2 of 5 (40%) *ERBB2* amplified CRC patients displayed concurrent oncogenic alterations; the first patient displayed multiple *subclonal* driver variants (*BRAF* V600E, *KRAS* Q61H, and a *CCDC6-RET* fusion) as well as *EGFR* ectodomain mutations (G465R, S464L) suggestive of the acquisition of several on- and off-target resistance mutations in response to standard-of-care cetuximab therapy while the second patient had a co-occurring *subclonal KRAS* G12R mutation (Figure 5, Supplementary Table 2). Recent cfDNA results from the HERACLES study (lapatinib plus trastuzumab in *ERBB2*-amplified metastatic CRC) found that the majority of non-responders (6/7) harbored concomitant *clonal RAS*/*RAF* gene mutations at baseline while *subclonal* RAS/RAF/PI3K-AKT pathway alterations emerged at progression after initial response or disease stabilization. The presence of *subclonal RAS*/*RAF* mutations co-occurring with *ERBB2* amplification in this cohort of CRC patients begs the question if targeted HER2 therapy may still achieve benefit and/or if a combination approach might be warranted (27, 28). One *ERBB2* amplified GC patient had a co-occurring *subclonal PIK3CA* E545G variant (1/6; 16.6%); one other had an atypical *KRAS* V14I loss-of-function mutation although its clinical significance remains to be elucidated. This is in stark contrast to non-GI cancers where half display co-occurring oncogenic alterations (Figure 5).

The most common co-occurring alterations observed in *ERBB2* amplified GI patients were copy number gains in other genes, including other receptor tyrosine kinases (ex. *MET, EGFR, FGFR1*) and cell cycle genes (ex. *CCNE1, CDK4/6*). Many of these can, in principle, serve as bypass mechanisms of resistance to not only standard-of-care but HER2-directed therapies and are potentially targetable. Understanding the genomic landscape of various GI tumor types or in individual patients may facilitate the determination of optimal combination therapies in specific genetic contexts. For instance, two-thirds of *ERBB2*-positive patients in the CRC cohort had evidence of *EGFR* co-amplification providing potential rationale for combining a HER2 targeted agent with cetuximab/chemotherapy in these patients. Alternatively, treatment with a dual HER2/EGFR inhibitor, such as lapatinib, may be warranted in this scenario.

Here we presented two patient case reports of patients with metastatic GC with high-level focal *ERBB2* amplifications as detected by cfDNA. These reports provide examples of how cfDNA testing can inform treatment decision both at diagnosis and at progression by (1) identifying the actionable alteration/capturing intertumoral heterogeneity and (2) confirming a lack of co-occurring mutations that can confound response to targeted therapy. Neither patient had evidence of alternative driver alterations in the plasma and both benefited from combined trastuzumab + chemotherapy therapeutic regimens (Figures 6A,B). Critically, *ERBB2* amplification and overexpression was identified earlier in the first patient's clinical journey and hence he experienced a partial and a more durable response to therapy. Unfortunately, the second patient had already failed several lines of therapy but remarkably still had disease stabilization and symptom improvement when treated with trastuzumab. In addition to these case reports, several others have been published demonstrating that patients with advanced cancers treated based on a positive cfDNA test result derive clinical benefit from the corresponding molecularly-targeted therapy (summarized in Table 1). An important consideration is that Asian patients may have differential sensitivity to specific drugs and combination therapy approaches. It has been reported that a subset of Asian patients with GC derived benefit from treatment with lapatinib and trastuzumab in the LOGiC trial, while this combination was not effective in Caucasian patients (33). Hence optimal treatment strategies may need to be independently established for patients from different geographical regions and racial/ethnic backgrounds.

**Table 1:** Summary of publications demonstrating clinical validity and utility of Guardant360® cell-free DNA test in gastrointestinal cancers.

	**Cancer type**	**Genomic target**	**ctDNA role in study**	**Tissue concordance**	**Therapeutic regimen**	**Key findings**
Hong et al. (29)	mCRC	*BRAF* V600E	Correlative	N/A	Vemurafenib + irinotecan + cetuximab	•35% (6/17) objective response rate | 88% (15/17) disease control rate •Median PFS 7.7 months •Near perfect correlation between Guardant360-detected and ddPCR-detected *BRAF*^V600E^ •*BRAF*^V600E^ ctDNA trends over time correlated with radiographic changes •ctDNA analysis identified mutations in genes reactivating MAPK signaling at progression
Montagut et al. (30)	mCRC	*EGFR* extracellular domain mutations	Exploratory secondary objective	N/A	Sym004 + investigators choice	•Sym004 did not improve overall survival in an *unselected* population of mCRC patients and acquired anti-EGFR resistance •Guardant360 analysis defined a triple negative subgroup (*RAS*/*BRAF*/*EGFR* ECD negative) with improved median OS (12.8 vs. 7.3 m)
Siravegna et al. (27)	mCRC	*ERBB2* (HER2) amplification	Correlative	98% (*ERBB2* amp detected in 51/52 samples)	Trastuzumab + lapatinib	•24% (8/32) objective response rate •Guardant360 identified *clonal RAS/RAF* mutations in 86% of primary resistance cases and acquired *subclonal RAS/RAF, ERBB2, EGFR, PIK3CA*, etc. mutations at progression
Kim et al. (13)	Gastric/GE	*ERBB2* (HER2) amplification (and others)	Treatment selection	N/A	Lapatinib + Capecitabine + Oxaliplatin	•Multiple parallel cohort, open-label, clinical trial using ctDNA-guided matched therapy when tissue was insufficient, or unobtainable for NGS •80% (4/5) objective response rate among *ERBB2* (HER2) amplified cases •67% (6/9) objective response rate including *ERBB2* amplified (4/5), *MET* amplified (1/1), *FGFR2* amplified (0/1), and *PIK3CA* mutant (1/2)
Pectasides et al. (14)	Gastric/GE	*ERBB2* (HER2) amplification (and others)	Correlative	85% concordance between cfDNA and metastases	FOLFOX or FOLFIRI + targeted agent when applicable	•Significant discordance between primary GE tumors and metastases based on tissue testing in 36% (10/28) of patients leading to treatment change in nine patients (32% of 28) •In five discordant cases, no actionable genomic alteration was detected in the primary, yet the metastasis and Guardant360 both revealed actionable copy number amps in *ERBB2* (2), *MET* (1), *EGFR* (1), or *FGFR2* (1)
Kim et al. (12)	Gastric/GE	*ERBB2* (HER2) amplification	Exploratory secondary objective	67% (6/9) among all; 86% (6/7) among responders	Lapatinib + Capecitabine + Oxaliplatin	•69% (22/32) objective response rate •Significant *ERBB2* (HER2) amp discordance, 40% (4/10), between primary tumor and metastasis based on tissue testing •Detectable *ERBB2* copy number amplification in plasma at baseline was predictive to the response (100% response rate) and changes in plasma-detected genomic alterations were associated with lapatinib sensitivity and/or resistance
Maron et al. (31)	Gastric/GE	*EGFR* amplification	Correlative	86% (6/7 *EGFR* amplification)	EGFR mAB ± chemotherapy	•Treatment details: 3 FOLFOX + ABT-806; 1 FOLFORI + cetuximab; 3 cetuximab •57% (4/7) objective response rate | 100% disease control rate •Median PFS 10.0 months •Serial ctDNA and tissue NGS identified mechanisms of primary and acquired resistance in all patients
Kim et al. (32)	Gastric/GE	TMB-high or PD-L1 >1% or MSI-high	Exploratory secondary objective	See key findings	Pembrolizumab	•Good concordance (*r*^2^ = 0.54) between ctDNA and tissue exome TMB with one outlier who had high ctDNA TMB/low tissue TMB; second tissue biopsy showed high TMB (i.e., tumor heterogeneity captures by ctDNA) •25% (15/61) objective response rate overall; a Guardant360 “digital” tumor mutation burden score in the top tertile predicted improved ORR (83 vs. 7.7%, *p* = 0.0014)

Nonetheless, an important advantage of cfDNA testing is the ability to capture both inter- and intratumoral heterogeneity to obtain a holistic view of the genomic landscape in primary and metastatic lesions. In the PANGEA trial of personalized antibodies for gastroesophageal patients, profiling of both primary and metastatic tumor tissue specimens led to treatment reassignment in almost a third of patients, many of which had HER2-positive metastases (14). Importantly, cfDNA and metastatic tissue genotyping results were 87.5% concordant while substantive discordance was seen between primary and metastatic tissue genotyping and between primary tissue and cfDNA genotyping results. Similarly, in a recent trial of pembrolizumab as salvage therapy in advanced GC patients, cfDNA analysis identified a tissue microsatellite stable (MSS)/tumor mutation burden (TMB)-low patient with pronounced intratumoral heterogeneity as being TMB-high. A second biopsy at the same site confirmed MSI/TMB-high status consistent with the cfDNA result, further highlighting that plasma testing allows for the enrichment and analysis of DNA shed from various heterogeneous tumor sites (32). Such inter- and intratumoral heterogeneity is certainly not limited to GC but is also observed in CRC and other cancer types. It has been reported that CNAs are the major source of tumor heterogeneity in CRC development (34). The same authors found that *ERBB2* amplification is acquired in 6–7% of metastases. Given that distal metastases are a major cause of morbidity in cancer patients, targeted treatment of the secondary lesion(s) is strongly advisable if not recommended, as in the case of breast cancer. cfDNA genotyping provides a non-invasive solution that can drive therapy selection in metastatic patients where tissue sampling multiple metastatic lesions is not practical or in some cases possible.

A limitation to our study is that the relative patient numbers by cancer type are skewed based on standard-of-care ordering practices. For instance, lung cancer patients are over-represented in the Guardant Health patient cohort given that testing for actionable alterations at diagnosis, or progression on a targeted therapy, is routine. In the same vein, we observed a lower prevalence of *ERBB2* amplification in this breast cancer patient cohort compared to that in a general population (3 vs. 10–15%, respectively) but a higher incidence of *ESR1* mutations (Supplementary Figure 3) associated with progression on hormone-based therapies. Another important limitation is the lack of clinically annotated Asian patient cfDNA samples (i.e., disease stage, lines of therapy, tissue test results, and clinical outcomes). Establishing the genomic landscape of *ERBB2* amplified Asian patients in more defined clinical contexts will be key in identifying and defining appropriate treatment strategies, and their sequencing, by disease stage and patient treatment history. While an impressively high degree of concordance (97.9%) has been described between cfDNA and tissue *ERBB2* amplification status of CRC patients enrolled in the HERACLES trial (27), a head-to-head comparison of tissue and blood-based test results in larger patient cohorts will be necessary to define and develop HER2 positivity criteria. Indeed, “low” expressers have been described to derive some clinical benefit from anti-HER2 agents such that a more extensive study will be necessary to define cut-offs, particularly in the absence of alternative therapies.

## Conclusion

Here we demonstrate, for the first time, that high level, focal *ERBB2* gene amplification, as identified by cfDNA testing, is a common event in Asian patients with advanced cancers. In particular, higher *ERBB2* incidence and CN gains were observed in GC and CRC patients in the absence of other oncogenic alterations, indicating that HER2 may be the dominant driver of tumor proliferation in those settings. Given its established role as an oncogene in certain contexts and the availability of HER2 antagonists, both approved and in clinical development, *ERBB2* amplification is an attractive therapeutic target. Comprehensive cfDNA testing represents a non-invasive method of assessing HER2 status in the metastatic disease setting. It not only captures inter- and intratumoral heterogeneity but allows for assessment of co-occurring somatic mutations and the identification of patients most likely to benefit from HER2-based therapeutic strategies.

## Data Availability

The datasets generated for this study are available on request to the corresponding author.

## Author Contributions

JL, AF, VR, and RL conceived and designed the study. AF and YS collected, assembled, and analyzed the data. AF, KB, VR, and RL interpreted the data. JL, SK, and K-MK treated the patients and provided clinical samples. AF, JL, and RL wrote the manuscript. All authors contributed to manuscript revision, read, and approved the submitted version.

### Conflict of Interest Statement

AF, YS, KB, VR, and RL are employees and shareholders of Guardant Health Inc. The remaining authors declare that the research was conducted in the absence of any commercial or financial relationships that could be construed as a potential conflict of interest.
