# Supplementary material for: Detection of ERBB2 (HER2) Gene Amplification Events in Cell-Free DNA and Response to Anti-HER2 Agents in a Large Asian Cancer Patient Cohort
Source: Front Oncol. 2019 Apr 4;9:212. doi: 10.3389/fonc.2019.00212 (PMC6458313; doi:10.3389/fonc.2019.00212)

Supplementary Material

Detection of *ERBB2* (HER2) gene amplification events in cell-free DNA and response to anti-HER2 agents in a large Asian cancer patient cohort

Jeeyun Lee^1*^, Aleksandra Franovic^2*^, Yukimasa Shiotsu^2^, Seung Tae Kim^1^, Kyoung-Mee Kim^3^, Kimberly C. Banks^2^, Victoria M. Raymond^2^, and Richard B. Lanman^2^

*** Correspondence:** Jeeyun Lee ([Jyunlee@skku.edu](mailto:Jyunlee@skku.edu)) and Aleksandra Franovic ([afranovic@guardanthealth.com](mailto:afranovic@guardanthealth.com))

# Supplementary Tables

## Supplementary Table 1. Cohort demographics for 469 unique patients from Asia.


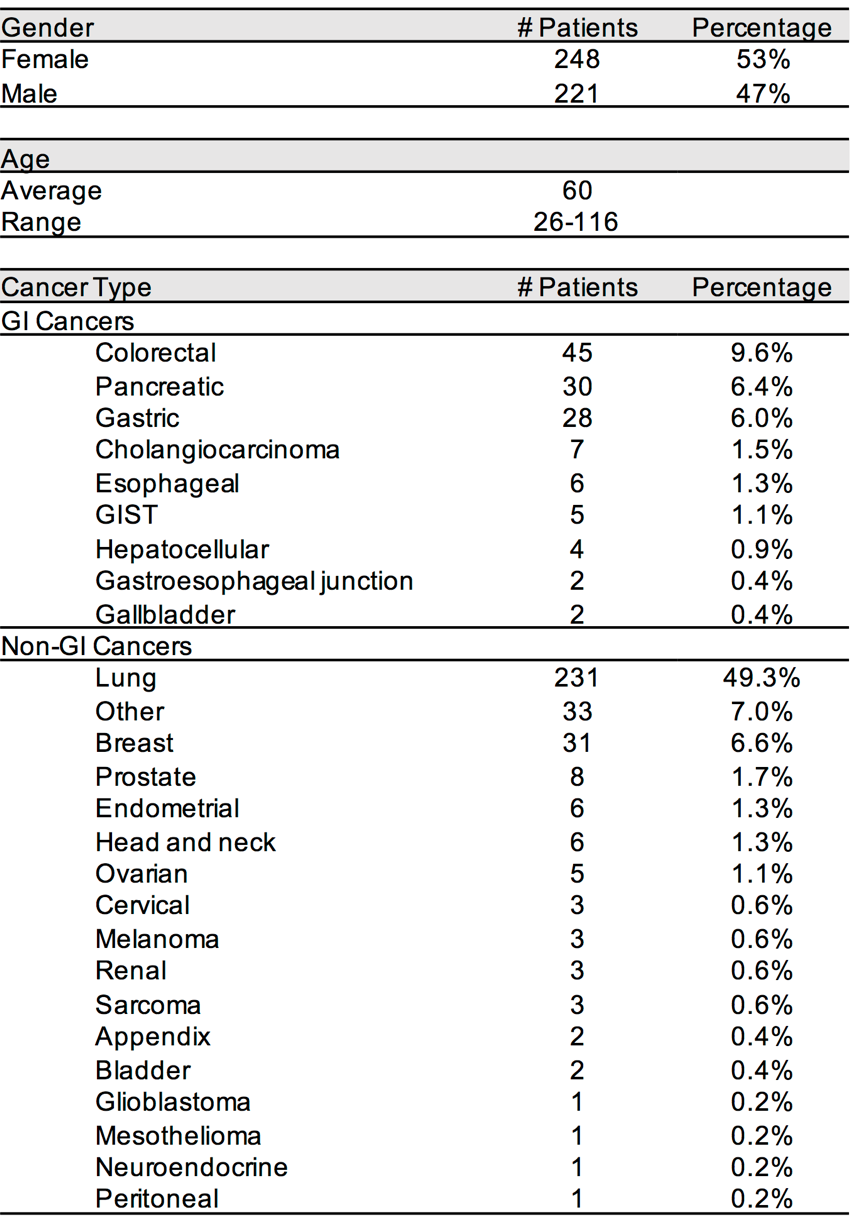


## Supplementary Table 2. Somatic variants detected by cfDNA testing in cohort samples.

Excel spreadsheet attached.

# Supplementary Figures

## Supplementary Figure 1. Percentage of all cfDNA tested samples by country.

## Supplementary Figure 2. Prevalence of *ERBB2* gene amplifications in Asian patient cohort by cancer type. Total number of *ERBB2* copy number amplification (CNA)-positive and – negative patients plotted. Prevalence is indicated above individual bars. Abbreviations: GI, gastrointestinal; GC, gastric cancer; CRC, colorectal cancer; HCC, hepatocellular cancer; PANC, pancreatic cancer; CC, cholangiocarcinoma; GIST, gastrointestinal stromal tumor; GEJ, gastroesophageal cancer; H&N, head and neck cancer.


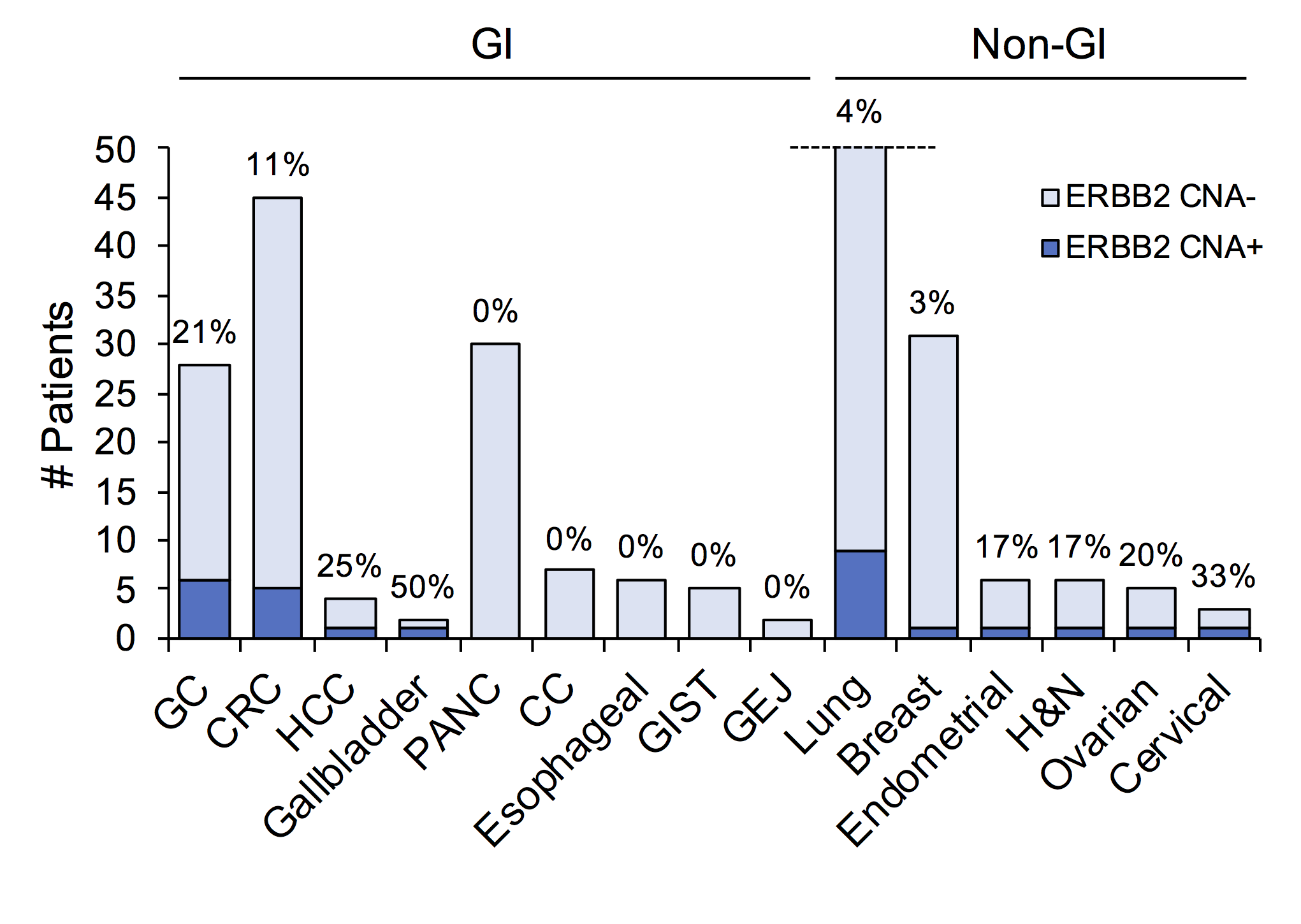


## Supplementary Figure 3. cfDNA genomic landscape among Asian breast cancer patient cohort (n=31). Number of patients exhibiting non-synonymous alterations plotted by gene. Most commonly altered genes include *TP53*, *PIK3CA*, and *ESR1*.


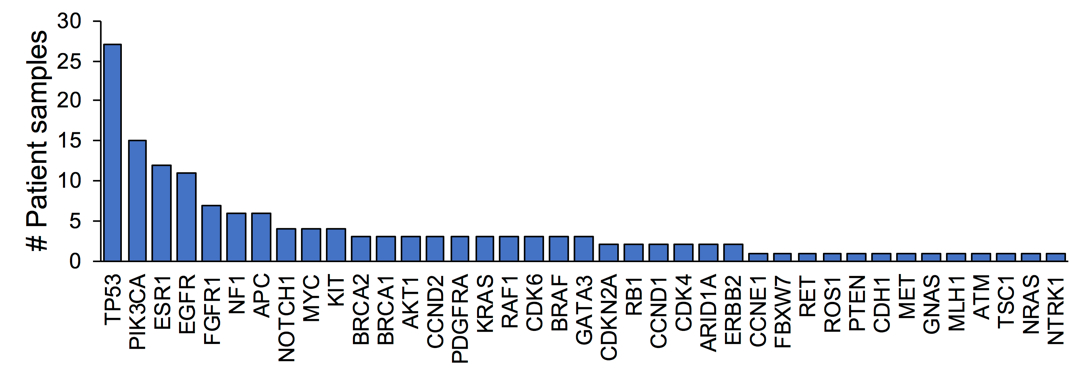

Supplement: Supplementary file 2 [file Data_Sheet_1.docx]
